# A rare subtype of lynch syndrome familial with co-mutation of EpCAM c.344T>C, MSH2 c.2744A>G, PMS2 c.1408C>T and APC c.5465T>A, case report and literature review

**DOI:** 10.3389/fgene.2025.1667899

**Published:** 2025-10-15

**Authors:** Guiyu Lu, Ting Pan, Cuidong Deng, Xiaoqian Wan, Zihan Wang, Tengyue Hu, Xianshuo Cheng, Jian Dong

**Affiliations:** ^1^ Department of General Practice, Zigong Fourth People’s Hospital, Zigong, Sichuan, China; ^2^ Department of Colorectal Surgery, The Third Affiliated Hospital of Kunming Medical University/Yunnan Tumor Hospital, Kunming, Yunnan, China

**Keywords:** lynch syndrome, epcam, MSH2, PMS2, APC, co-mutation, mosaicism, case report

## Abstract

**Background:**

Lynch syndrome (LS) is an autosomal dominant disorder caused by germline mutations in mismatch repair (MMR) genes or EpCAM, leading to various cancers, particularly colorectal cancer (CRC). EpCAM mutations account for approximately 1%–3% of LS cases, while co-mutations involving EpCAM and MSH2 are exceedingly rare. To date, co-mutations of EpCAM, MSH2 and PMS2 have not been reported in the literature.

**Case Presentation:**

This case reports a 25-year-old male diagnosed with adenocarcinoma of the ascending colon. His family history revealed eight cancer cases among 30 relatives across five generations, consistent with LS. Immunohistochemistry (IHC) of the tumor showed loss of EpCAM, MSH2 and MSH6 protein expression. Genetic testing of the proband’s tumor identified a novel large deletion affecting EpCAM exons 8-9 and MSH2 exons 1–16, likely pathogenic mutations disrupting MMR gene function. Whole-exome sequencing (WES) of peripheral blood from six family members, including the proband and his son, revealed co-mutations of EpCAM (c.344T>C), MSH2 (c.2744A>G), PMS2 (c.1408 C>T) and APC (c.5465T>A). Although public databases suggested these variants are benign or of uncertain significance (VUS), several *in silico* prediction tools and prior literature suggest potential pathogenicity. Notably, WES of the proband’s son’s peripheral blood also detected the same large deletions in EpCAM and MSH2, implying the presence of germline mosaicism and a possibly heightened early-onset cancer risk.

**Conclusion:**

This rare subtype of LS emphasizes the need for comprehensive genetic screening and may inform future strategies for early detection and management in LS families. Further studies are required to confirm these findings.

## Introduction

Lynch syndrome (LS), also known as hereditary nonpolyposis colorectal carcinoma (HNPCC), is the most common hereditary colorectal cancer (CRC) syndrome, accounting for approximately 2%–4% of all CRCs. LS is an autosomal dominant disorder caused by germline mutations in mismatch repair (MMR) genes (MLH1, MSH2, MSH6, PMS2) or the epithelial cell adhesion molecule (EpCAM) gene (Vasen et al., 2010). These mutations lead to MMR deficiency (dMMR) and high microsatellite instability (MSI-H), resulting in genomic instability and increased tumor susceptibility. In addition to CRC, LS is associated with increased risks of endometrial, gastric, ovarian, and brain cancers. The spectrum and magnitude of cancer risk among LS families vary depending on which specific MMR or EPCAM gene is mutated.

Population-based prospective data further highlight gene-specific penetrance. According to the Prospective Lynch Syndrome Database (PLSD), the cumulative cancer risks by age 70 are estimated at 71.9% for MLH1, 74.5% for MSH2, 46.3% for MSH6, and 21.7% for PMS2 mutation carriers (Vasen et al., 2007; Prospective Lynch Syndrome Database, 2025). CRC risk is particularly high in MLH1 and MSH2 carriers (48.2% and 43.7%, respectively), whereas endometrial cancer (EC) risk is most elevated in female MSH2 and MSH6 mutation carriers (46% and 41%, respectively). Although brain tumors are relatively uncommon in LS, their relative risk is highest in MSH2 carriers (3.7%).

EpCAM deletions are rare, accounting for only 1%–3% of LS cases (Tiwari et al., 2016). A well-established mechanism is that 3′EpCAM deletions can lead to MSH2 promoter hypermethylation, thereby silencing MSH2 expression and triggering dMMR (Niessen et al., 2009). In a cohort of 41 LS families, EpCAM deletion carriers had a cumulative CRC risk of 75% by age 70, which was higher than that of MSH6 carriers (50%) and similar to that of MSH2 (77%) and MLH1 mutation carriers (79%) (Kempers et al., 2011). Notably, female carriers with EpCAM-MSH2 co-deletions showed a markedly increased EC risk (55%) compared with EpCAM-only deletions (12%) (Kempers et al., 2011). These findings suggest that *EpCAM-related alterations not only confer substantial CRC risk but also modify extracolonic cancer susceptibility depending on their interaction with MSH2*.

At the molecular level, MSH2 with MSH6 forms MutSα complex that recognizes DNA mismatches, while PMS2 with MLH1 forms MutLα complex that executes the excision and repair step of MMR (Li, 2008-01). This division of labor highlights their complementary roles in genomic maintenance. APC, a gatekeeper of the Wnt/β-catenin pathway, initiates colorectal tumorigenesis (Kinzler and Vogelstein, 1996-10). In this context, MMR deficiency may accelerate APC-related mutational events, while APC loss in a dMMR background can further lower the threshold for malignant transformation. Thus, concurrent EpCAM/MSH2, PMS2, and APC alterations provide a biological rationale for synergistic tumorigenesis.

Despite these insights, most prior studies focus on single-gene mutations or EpCAM–MSH2 co-deletions, often limited to case reports or small series. Important knowledge gaps remain: (i) how concurrent alterations spanning multiple loci (e.g., EpCAM, MSH2, PMS2, and APC) jointly shape penetrance, tumor spectrum, and age at onset; (ii) whether point mutations and structural variants interact to produce tissue-specific silencing and phenotype modification; and (iii) to what extent current surveillance guidelines, which are organized on a gene-by-gene basis, adequately capture multi-locus constellations.

To address these gaps, we report a LS family harboring a previously undocumented co-mutation subtype: EpCAM c.344T>C, MSH2 c.2744A>G, PMS2 c. 1408 C>T and APC c.5465T>A. Notably, we identified novel large fragment deletions spanning EpCAM exons 8–9 and MSH2 exons 1–16 in the proband’s CRC tissues. This case underscores the complexity of LS genetics and highlights the need for comprehensive evaluation incorporating both point mutations and structural variations, to better inform clinical management and surveillance strategies.

## Case presentation

### Proband’s clinical details

**FIGURE 1:**
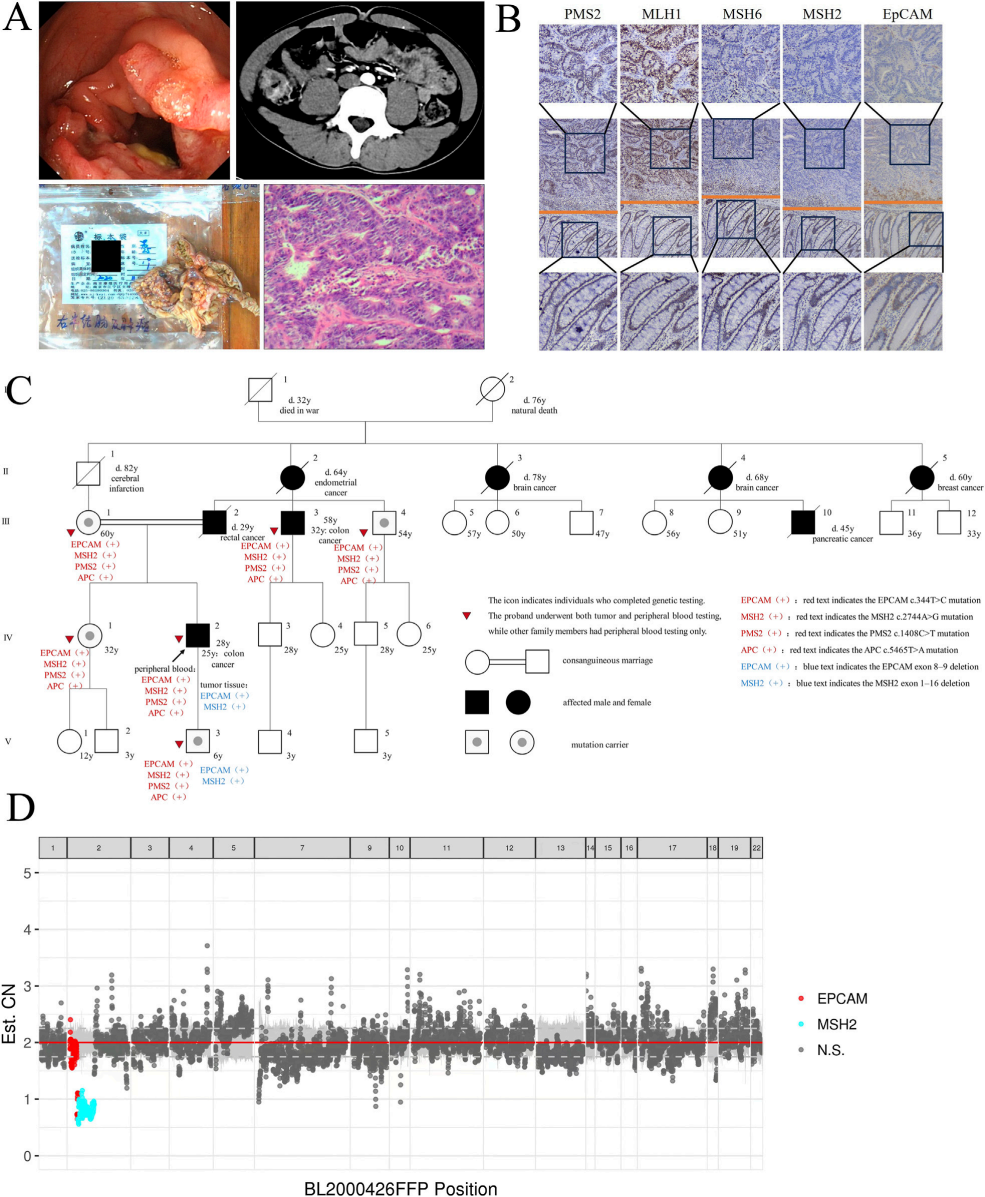
Clinical and molecular analysis of the LS family. **(A)** Colonoscopy figure, abdominal CT scan, surgical resection of ascending colon tumor specimen and HE staining of CRC tissues of the proband (IV-2). **(B)** IHC Staining of EpCAM and MMR Protein in the Proband’s CRC Tissue. Columns displayed from top to bottom: tumor tissue (400×), tumor-normal boundary (100×) and normal tissue (400×), respectively. **(C)** Pedigree structure of the LS family. Squares denote males, circles denote females, solid symbols represent cancer cases, the arrow indicates the proband, slashes mark deceased individuals with age at death, and tumor types are listed below each symbol. Solid circles in squares indicate mutation carriers. Consanguineous marriages are represented by double horizontal lines. **(D)** Copy Number Variation (CNV) Analysis of Proband’s CRC Tissues. The plot shows CNVs across genomic positions in tumor tissue. Red dots indicate CN loss in EpCAM, cyan dots indicate CN loss in MSH2, and gray dots represent non-significant regions.

In October 2020, a 25-year-old male presented with a 1-month history of right lower abdominal pain and localized tenderness. Colonoscopy revealed an annular elevation in the ascending colon (Figure 1A), confirmed as adenocarcinoma by histopathology. CT indicated asymmetric thickening of the ascending colon wall (Figure 1A). In November 2020, he underwent 3D laparoscopic right hemicolectomy. Postoperative pathology revealed moderately to poorly differentiated adenocarcinoma (pT3N0M0, stage IIA) (Figure 1A). IHC showed loss of EpCAM, MSH2, and MSH6 expression, with preserved MLH1 and PMS2 (Figure 1B). Adjuvant chemotherapy was not administered based on guidelines.

As of the 56-month follow-up in May 2025, regular test confirmed disease-free survival. Genetic counseling addressed his concerns about hereditary cancer risk, especially for his son.

### Family history

Several family members have been diagnosed with cancer (Figure 1C), including the proband’s father (III-2, 29-year-old, rectal cancer), uncle (III-3, 32-year-old, colon cancer), grandmother (II-2, 64-year-old, EC), great-aunts (II-3, 78-year-old, and II-4, 68-year-old, brain cancer; II-5, 60-year-old, breast cancer), and cousin (III-10, 45-year-old, pancreatic cancer).

### Genetic testing

#### Tumor tissue genetic testing

**FIGURE 2:**
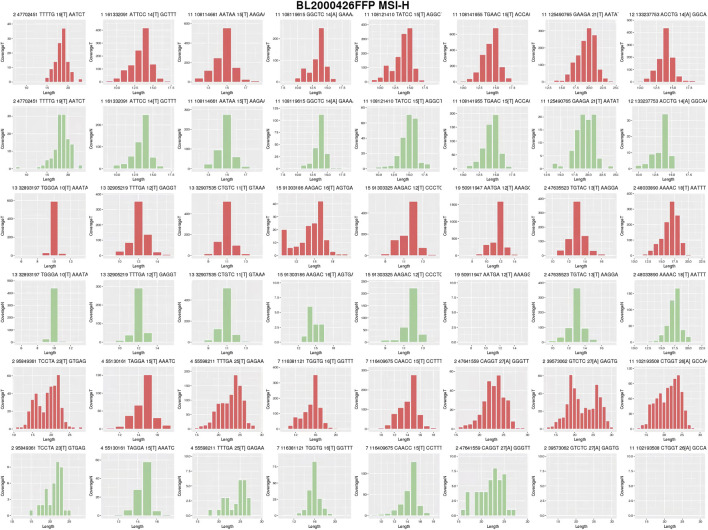
MSI analysis of Proband’s CRC Tissues. Tumor (red) and normal (green) distributions are shown. The proportion of unstable loci was calculated using cumulative distribution shift analysis. The instability rate was 30.43%, exceeding the 20% threshold for MSI-H.

Next-generation sequencing (NGS) (Figure 1D) of the proband’s CRC tissue identified novel large deletions in EpCAM (exons 8–9) and MSH2 (exons 1–16), not previously documented in the International Society for Gastrointestinal Hereditary Tumors (InSiGHT) database. To date, only three similar EpCAM-MSH2 co-deletions have been reported (Table 1): case 1 (Sekine et al., 2017) (EpCAM exon nine to MSH2 exon 1–2), case 2 (Salman et al., 2018) (EpCAM exon 8-9 and MSH2 exon 1–8), case 3 (Huang et al., 2022) (EpCAM exon 1-9 to MSH2 exon 1–6). These structural variants are pathogenic, disrupting MSH2 function via epigenetic silencing or gene interruption. Microsatellite instability (MSI) analysis revealed a 30.43% instability rate (MSI-H) (Figure 2).

**TABLE 1:** Six cases of LS with co-mutation of EpCAM and MSH2.

No.	Case 1 (Sekine et al., 2017)	Case 2 (Salman et al., 2018)	Case 3 (Huang et al., 2022)	Case 4 (Stoffel et al., 2009)	Case 5 (Silva et al., 2009)	Our Case
Basic Information	44-year-old female	38-year-old male	45-year-old female	54-year-old female	9-year-old female	25-year-old male
Cancer History	Colon cancer at 39, 43, and 44 (poorly differentiated mucinous adenocarcinoma)	Synchronous poorly differentiated adenocarcinoma of the cecum, appendix, and ileocecal area, with retroperitoneal and vertebral metastasis	Moderately differentiated endometrial adenocarcinoma with post-surgical vaginal and lymph node metastasis	Sigmoid colon cancer at 37, ascending colon cancer at 41, endometrial cancer at 42, duodenal adenocarcinoma at 45, breast cancer at 52	Rectal cancer, multiple colonic polyps	Moderately to poorly differentiated ascending colon adenocarcinoma at 25
IHC	MSH2 nuclear (−), cytoplasm (+); MSH6 (−) EPCAM (+)、MLH1 (+), PMS2 (+)	MSH2 (−)、EPCAM (−) MLH1 (+)、PMS2 (+)	MSH2、MSH6 (−) EPCAM (+)、MLH1 (+)、PMS2 (+)	MSH2 (−)、MSH6 (−) EPCAM (+)、MLH1 (+)、PMS2 (+)	MSH2 (−)、MSH6 (−) EPCAM (+)、MLH1 (+)、PMS2 (+)	MSH2 (−)、MSH6 (−)、EPCAM (−) MLH1 (+)、PMS2 (+)
Genetic Testing	Deletions in EpCAM exon 9 and MSH2 exons 1 and 2, EPCAM-MSH2 fusion	Deletions in EpCAM exon 1–9 and MSH2 exons 1–6	Deletions in EpCAM exons 8–9 and MSH2 exons 1–8	EpCAM full gene and MSH2 exons 1–7 duplication, heterozygous deletions on chromosomes 20 and X	Deletions in EpCAM exons 8–9. MSH2 missense mutation c.2075G>T	EPCAM c.344T>C, MSH2 c.2744A>G, APC c.5411T>A (germline) Deletions in EpCAM exons 8–9 and MSH2 exons 1–16 (CRC tissue)
Family History	Mother: colon cancer (40s), endometrial cancer (50s); sister: colon cancer (30s), ovarian cancer (40s)	Father: colon cancer, skin cancer	First-degree relatives: endometrial and colorectal cancers; second-degree relatives: gallbladder and gastric cancers	Two sisters: colon cancer (43s, 40s); another sister: pancreatic cancer (47s); nephew: colon cancer (27s); brother: colon cancer (55s)	First-degree relatives: no cancer; paternal second-degree: early-onset colon and gastric cancer; maternal second- and third-degree: uterine, gastrointestinal, renal, and brain cancers	Father: rectal cancer (29s); uncle: colon cancer (32s); grandmother: endometrial cancer (64s); two maternal aunts: brain cancer (78s, 68s); another maternal aunt: breast cancer (60s); cousin: pancreatic cancer (45s)
Pathogenic Mechanism	EpCAM and MSH2 deletions cause EpCAM-MSH2 fusion and MMR deficiency	EpCAM and MSH2 exon deletions, MMR deficiency	EpCAM and MSH2 exon deletions, MMR deficiency	EpCAM-MSH2 duplication, MMR deficiency; LOH on chromosomes 20 and X, increasing cancer risk	EpCAM mutation silences MSH2 in epithelial cells, increasing epithelial tumor risk	EpCAM and MSH2 mutations with large deletions, MMR deficiency; mosaicism increase cancer risk; unknown APC mutation may promote tumorigenesis

#### WES of peripheral blood in family members

WES of peripheral blood from six family members (proband IV 2, III 1, III 3, III 4, IV 1, V 3) identified mutations in EpCAM (NM_002354.3:c.344T>C, p.Met115Thr), MSH2 (NM_000251.3:c.2744A>G, p.Gln915Arg), PMS2 (NM_000535.7:c.1408C>T, p.Pro470Ser) and APC (NM_000038.6:c.5465T>A, p.Val1822Asp) in all (Figure 1C; Table 1). Remarkably, the proband’s son (V-3) also carried the same EpCAM and MSH2 deletions as the proband’s tumor tissue.

## Discussion

LS is an autosomal dominant syndrome with high cancer susceptibility, as 54%–61% of patients develop a second primary tumor and 15%–23% develop three or more (Stoffel et al., 2009). Diagnosis is based on family history (Amsterdam II Criteria, the Revised Bethesda Guidelines, and the Chinese Criteria) and confirmed by genetic testing of MMR, EpCAM, and BRAF V600E. Among LS families, MSH2 mutations account for ∼50%, MLH1 for 30%–40%, MSH6 for 7%–10%, and PMS2 for <5% (Silva et al., 2009).

Co-mutations involving EpCAM and MSH2 are rare. A cohort study of 41 LS families, including 194 EpCAM deletion carriers, identified 42 patients with both EpCAM and MSH2 co-deletions (Kempers et al., 2011). Additionally, only five case reports document LS patients with EpCAM and MSH2 co-mutations (Table 1).

- Case 1 describes a 44-year-old woman with three episodes of CRC, whose family history includes her mother (CRC and EC) and sister (CRC and ovarian cancer) (Sekine et al., 2017).
- Case 2 reports a 38-year-old man with three synchronous intestinal cancers, whose father had a history of intestinal and skin cancers (Salman et al., 2018).
- Case 3 involves a 45-year-old woman with metastatic EC, where first-degree relatives had EC and CRC, and second-degree relatives had gallbladder and gastric cancers (Huang et al., 2022).
- Case 4 concerns a 54-year-old woman who developed CRC three times, along with EC and breast cancer. Her family history includes two sisters who died of CRC, another sister who died of pancreatic cancer, and both a brother and nephew diagnosed with CRC at ages 55 and 27, respectively (Pirini et al., 2019).
- Case 5 is a 9-year-old female with rectal cancer and multiple colonic polyps. Her parents have no cancer history, but paternal relatives have early-onset CRC and gastric cancers. Several maternal relatives have malignancies involving the uterus, gastrointestinal tract, kidneys, and central nervous system (Li-Chang et al., 2013).

To our knowledge, co-mutations involving EpCAM, MSH2, PMS2 and APC have not been reported. We present an LS family in which all six tested members carried EpCAM c.344T>C, MSH2 c.2744A>G, PMS2 c.1408C>T, and APC c.5465T>A co-mutations, with the proband’s CRC tissue showing large deletions of *EpCAM* (exons 8–9) and *MSH2* (exons 1–16). Tumor testing corroborated the sequencing results: IHC of the proband’s tumor showed loss of EpCAM, MSH2, and MSH6 with retention of MLH1 and PMS2, consistent with MSH2-pathway deficiency; MSI testing demonstrated 30.43% unstable loci (MSI-H). These findings confirm dMMR and link EpCAM-associated MSH2 disruption to the observed immunophenotype (Figures 1B,E). Tumor testing was limited to the proband, restricting assessment across relatives.

In this family, eight members developed cancers at ages 25–32, including three CRCs before 40 (youngest 25), two brain cancers, one EC, one breast cancer, and one pancreatic cancer. Among the six carriers tested, two (33%) had developed cancer while four (67%) remained unaffected, illustrating incomplete penetrance. This rate is lower than PLSD estimates (∼45% for MLH1/MSH2 carriers by age 70), likely reflecting the relatively young age of unaffected carriers in this family (Prospective Lynch Syndrome Database, 2025). Compared with published cohorts, this family shows unusually early CRC onset and excess brain cancer. CRC onset was earlier than reported by the German Consortium (42–69 years for MSH6, 61–66 years for PMS2, ∼44 years for MLH1/MSH2) and PLSD (49–50 years for MSH2) (Gupta et al., 2019). The five reported EpCAM/MSH2 co-mutation cases also showed early CRC (9–54 years), and Danish data reported brain cancers in 14% of LS families (mainly MSH2) (Therkildsen et al., 2015), compared to 3.7% in PLSD male MSH2 carriers. In contrast, brain tumors occurred in 25% of affected members here. Compared to recent findings (Niessen et al., 2009; Markowitz and Bertagnolli, 2009), the co-mutation of EpCAM, MSH2, PMS2 and APC might be a pathogenic factor, though statistical validation is lacking. While most tumors were single primaries, metachronous cancers cannot be excluded. The strong family history facilitated early CRC detection by colonoscopy in the proband and his uncle, leading to favorable outcomes with standard management.

Genetic testing revealed multiple germline mutations together with large deletions in EpCAM and MSH2, suggesting a hereditary multi-tumor etiology. Review of public databases (NCBI, MGeND, LOVD, OncoKB; accessed July 2025) classified EpCAM c.344T>C and APC c.5465T>A as benign, PMS2 c.1408 C>T as benign or neutral, and MSH2 c.2744A>G as VUS. Based on ACMG/AMP criteria (Richards et al., 2015), EpCAM c.344T>C and APC c.5465T>A fulfill BA1/BS1 owing to high allele frequency in gnomAD (Karczewski et al., 2020), and BP4 given consistent benign predictions. PMS2 c.1408C>T shows ∼35–40% frequency in gnomAD and 1,000 Genomes, meeting BA1 and BP4. In contrast, MSH2 c.2744A>G shows very low population frequency (<0.01) and inconsistent *in silico* results, thus remains VUS. Overall, these mutations appear unlikely to drive cancer (Table 2), though discordant predictions (e.g., MSH2 c.2744A>G was classified as pathogenic by SIFT) underscore the limitations of *in silico* tools and the need for functional validation.

**TABLE 2:** Clinical database annotations, in silico predictions, conservation analyses, and acmg/amp classifications of four mutations.

Mutation Gene	EpCAM (NM_002354.3:c.344T>C, p.Met115Thr)	PMS2 (NM_000535.7:c.1408C>T, p.Pro470Ser)	MSH2 (NM_000251.3:c.2744A>G, p.Gln915Arg)	APC (NM_000038.6:c.5465T>A, p.Val1822Asp)
Exon	3	11	16	16
ClinVar Significance	Benign	Benign	Not recorded	Benign
MGeND Significance	Benign	Benign	Not recorded	Benign
LOVD Significance	Benign	Benign	Not recorded	Benign
OncoKB Significance	Not recorded	Likely Neutral	Not recorded	Not recorded
Pop Freq (gnomAD)	0.51515	0.37107	Not recorded	0.82099
CADD Prediction	0.238	0.089	0.021	13.24
MetaSVM Prediction	T	T	T	T
MetaLR Prediction	T	T	T	T
SIFT Prediction	T	T	D	T
Polyphen-2 Prediction	B	B	B	B
MutationTaster Prediction	P	P	P	P
GERP++RS	1.71	0.572	−3.02	3.59
phastCons (20-way)	0.815	0	0	0.991
Related Articles	6	1	3	13
Detailed Article List	Supplementary Table S1	The PMS2 c.1408C>T was detected at 100% allele frequency across replicates in an MSI-stable tumor with no other pathogenic PMS2 alterations. Lacking functional data, it is classified as likely benign or of uncertain significance	Supplementary Table S1	Supplementary Table S1
ACMG/AMP Classification	Benign (BA1/BS1, BP4)	Benign (BA1, BP4)	VUS (conflicting *in silico*; lacking functional evidence)	Benign (BA1/BS1, BP4)

Mutations are annotated using HGVS, nomenclature. Clinical databases (ClinVar, MGeND, LOVD, OncoKB) assess clinical and genetic relevance. “Benign”: no significant disease association; “Not recorded”: absent from major clinical databases, potentially classifying it as a VUS, pending further evidence; “Likely Neutral”: minimal or no impact on protein function and low oncogenic potential. Pop Freq reflects allele frequency from gnomAD (>1% suggests a common mutation; “Not recorded” indicates missing data). CADD: Scores >10 indicate potential deleteriousness, with ≥20 marking the top 1% most deleterious mutations. “Tolerated (T)”: no significant functional impact and likely benignity. “Deleterious (D)”: potential pathogenicity; “Tolerated (T)”: minimal impact on protein function, likely benignity. “Benign (B)”: unlikely functional effect. “Polymorphism (P)”: a common genetic mutation unlikely to cause disease. GERP++ RS (≥2.0) and phastCons (20-way, ≥0.9) measure evolutionary conservation, indicating functional importance.

Despite the overall benign or uncertain classification, several reports suggest possible biological effects of these mutations (Supplementary Table S1). EpCAM c.344T>C may weaken protein folding, thereby promoting cervical cancer progression (Hu et al., 2012; Sankpal et al., 2021), enhance tumor cell stemness to facilitate breast cancer growth (Jiang et al., 2011), and impair EpCAM’s inhibitory effect on cathepsin L, increasing the invasiveness of non-small cell lung cancer (Yang et al., 2014). APC c.5465T>A, though not disrupting the Wnt/β-catenin signaling pathway, may still promote colorectal adenoma formation. Wallis’s report indicates that this mutation could disrupt β-catenin regulation, leading to intestinal cancer or adenoma formation (Wallis et al., 1999). Moreover, Theodoratou et al. noted that this mutation might alter the response to environmental factors through Wnt pathway modulation, thus increasing CRC risk (Theodoratou et al., 2008). There is currently no evidence linking PMS2 c.1408 C>T to tumor development and progression (Yurgelun et al., 2017), and the significance of MSH2 c.2744A>G remains unclear. Overall, while ACMG/AMP classification favors benign or VUS categories, subtle functional effects and gene-environment interactions cannot be excluded, highlighting the need for further validation.

In this family, sequencing of six members revealed EpCAM, MSH2, PMS2 and APC co-mutations associated with early-onset CRC. These co-mutations may modify CRC risk under specific exposures. We employed multiple prediction systems to assess the clinical significance of each mutation. Only Polyphen-2 and MutationTaster classified these mutations as benign, while most other systems classified them as potentially benign. The CADD prediction system identified APC c.5465T>A as likely pathogenic, while SIFT prediction system classified MSH2 c.2744A>G as pathogenic (Table 2). Therefore, current prediction tools cannot fully capture the biological impact of co-mutations, underscoring the need for functional validation.

The unusual phenotype may reflect epistasis, as co-existing EpCAM, MSH2, PMS2, and APC mutations could interact across MMR and Wnt/β-catenin pathways. 3′ EpCAM deletions can silence MSH2, establishing an MMR-deficient background, and animal models show that MSH2 deficiency synergizes with APC loss to accelerate intestinal tumorigenesis (Reitmair et al., 1996).

V-3, a 6-year-old child, warrants attention as WES revealed EpCAM, MSH2, PMS2 and APC co-mutations along with large deletions in EpCAM (exons 8–9) and MSH2 (exons 1–16), paralleling his father’s CRC tissue and suggesting possible germline mosaicism. Mosaicism is increasingly recognized in cancer (Ogawa et al., 2022) but may be missed by blood-based genetic testing, complicating LS diagnosis and genetic counseling. Case reports have documented mosaic MSH2 mutations in Lynch (-like) syndromes—for example, Erell Guillerm et al. detected a low-frequency MSH2 mutation in tumor tissue but absent in blood, which was confirmed as germline mosaicism in her daughter (Guillerm et al., 2020), while Pastrello et al. described somatic mosaicism with uneven mutation distribution across tissues (Pastrello et al., 2009). Reviews emphasize that mosaic events are underdetected in LS with blood-only testing (Jansen and Goel, 2020), supporting multi-tissue or tumor-first approaches. Additional proband tissue could clarify this but was unavailable. Alternatively, recurrent EpCAM 3′ deletions with shared haplotypes in Dutch LS families (Kuiper et al., 2011-04) suggest ancestral inheritance as another plausible explanation for the clustering of EpCAM, MSH2, PMS2, and APC mutations in this pedigree.

LS family members carrying mutations require ongoing surveillance. According to current guidelines (Vasen et al., 2010), MMR mutation carriers should undergo colonoscopy every 1–2 years starting at age 20–25, or 2–5 years before the earliest CRC in the family. Based on the 25-year earliest onset, surveillance should begin by age 20, and potentially earlier for young carriers. Given the 25% incidence of brain cancer, annual neurological evaluations are warranted, with brain MRI performed when clinically indicated. Female carriers should also undergo endometrial and ovarian cancer surveillance, such as annual transvaginal ultrasound or endometrial biopsy from age 30–35. At the same time, cascade testing of at-risk relatives is essential to identify asymptomatic carriers and enroll them in surveillance programs at an appropriate age, thereby reducing cancer risk through early detection and intervention. This strategy facilitates personalized risk communication and supports informed decision-making for long-term management within families.

Constitutional mismatch repair deficiency (CMMRD) is a rare hereditary cancer syndrome caused by biallelic pathogenic MMR mutations, leading to childhood tumors in the brain, gastrointestinal tract, and blood. PMS2 mutations are the most predominant causative genes (Mar et al., 2025). Genetic testing in V-3 identified a heterozygous MSH2 mutation without biallelic mutations or CMMRD symptoms (e.g., early cancers, café-au-lait spots) (Wimmer and Kratz, 2010; Wimmer et al., 2014). V-3’s heterozygous status elevates LS-related cancer risk, distinct from CMMRD’s biallelic-driven childhood cancer predisposition (Wimmer et al., 2014). Wimmer et al. noted that CMMRD leads to early malignancies, whereas LS typically manifests later (Wimmer et al., 2014). Therefore, V-3 requires LS-specific surveillance, not CMMRD’s pediatric protocols. For offspring inheriting germline mosaic mutations, LS-related cancer screening is advisable before adulthood, as the youngest CRC patient identified was only 9 years old in published case reports (Li-Chang et al., 2013). Further testing of more samples in this family is essential, as the results hold significant importance for management and disease prediction.

### Limitations

This study has several limitations. First, the sample size and potential selection bias may limit the generalizability of our findings. Consanguinity within families may increase the frequency of rare mutations, complicating interpretation of pathogenicity. Second, the lack of functional validation reduces the certainty of our mutation classification, as *in silico* predictions cannot fully capture biological effects. Third, due to resource constraints, we were unable to conduct additional tissue analyses to confirm mosaicism or to perform comprehensive WES re-evaluations, thus limiting our insights into copy number alterations. Future studies with larger, multicenter cohorts, coupled with functional assays and multi-tissue analyses, are essential to overcome these limitations and provide more definitive evidence.

## Conclusion

This case report describes a LS family harboring a rare co-mutation of EpCAM c.344T>C, MSH2 c.2744A>G, PMS2 c. 1408C>T and APC c.5465T>A. Notably, we identified pathogenic large fragment deletions in EpCAM (exons 8–9) and MSH2 (exons 1–16) in the proband’s CRC tissue for the first time. These findings expand the LS genetic spectrum and highlight the complexity of interpretating co-mutations. Despite databases suggest these point mutations are benign or VUS, bioinformatic predictions and prior reports suggest possible functional relevance. Given these uncertainties, conclusion should be regarded as preliminary. Future research should include development of cellular or animal models to dissect the synergistic effects of these alterations, systematic exploration of mosaicism and founder effects, and integration of functional assays with genomic profiling. Clinically, surveillance and cascade testing remain essential, and therapeutic implications, including immunotherapy responsiveness in dMMR, warrant further investigation.

## Data Availability

The datasets generated and analyzed for this study are not publicly available due to patient privacy restrictions but are available from the corresponding author upon reasonable request.
